# Verses

**Published:** 1906-08

**Authors:** 


### VERSES.

### Iced Water Dangerous.

Full many a man both young and old
Has gone to his sarcophagus
By pouring water icy cold
Adown his hot œsophagus.

*Exchange.*

---

### Contentment.

Better than gold is a heart where contentment
Scatters its sunshine to lighten and bless,
Treading its path with no thought of resentment,
E'en though than others its share may be less.

*M. C. Brown.*

---

### "Epigram.

"Cries logical Bobby, to Ned, will you dare
A bet, which has most legs a mare or NO MARE?
A mare, to be sure, replied Ned with a grin:
And fifty I'll lay, for I'm certain to win.
Quoth Bob, you have lost, sure as you are alive;
A mare has but four legs, and NO MARE HAS FIVE.

*"Funnidos."*

